# Can mac-2 binding protein glycosylation isomer serve as a biomarker for predicting pulmonary arterial pressure and pulmonary hypertension in systemic sclerosis?

**DOI:** 10.55730/1300-0144.5984

**Published:** 2025-02-18

**Authors:** Rıdvan MERCAN, Dilara BULUT GÖKTEN, Sonat Pınar KARA, Neslihan MELİK ÜZÜM, Savaş GÜZEL

**Affiliations:** 1 Department of Rheumatology, Faculty of Medicine, Tekirdağ Namık Kemal University, Tekirdağ, Turkiye; 2 Department of Internal Medicine, Faculty of Medicine, Tekirdağ Namık Kemal University, Tekirdağ, Turkiye; 3 Department of Biochemistry, Faculty of Medicine, Tekirdağ Namık Kemal University, Tekirdağ, Turkiye

**Keywords:** Systemic sclerosis, galectin-3, progressive systemic sclerosis

## Abstract

**Background/aim:**

This study aimed to explore the role of Mac-2 binding protein glycosylation isomer (M2BPGi) serum levels as a biomarker that could contribute to understanding organ involvement and the overall disease process in systemic sclerosis (SSc).

**Materials and methods:**

The cross-sectional study examined 108 patients with SSc. Seventy-two people were included in the control group. Demographic and clinical characteristics of the patients, laboratory and radiological findings, pulmonary function tests and echocardiography results, and presence of pulmonary hypertension (PHT) based on echocardiographic evaluation were recorded. Venous blood samples of 5 mL were collected from individuals. Human M2BPGi levels in the samples were measured using a specific kit.

**Results:**

There was no significant difference between the M2BPGi levels in the patient (median = 4749.69 pg/mL, mean = 5351.75 ± 2483.97) and the control group (median = 4638.07, mean = 4611.86 ± 1333.15) (p = 0.071). Considering pulmonary arterial pressure (PAP) status, the average M2BPGi level in the normal PAP group was 5898.15 ± 2555.61 pg/mL, while it was 4258.96 ± 1973.08 pg/mL in the increased PAP group. The difference between these groups was statistically significant (p: 0.021). Examining PHT status, the average M2BPGi level was 5942.01 ± 2579.14 pg/mL in the group without PHT, decreasing to 4264.44 ± 1917.63 pg/mL in the group with PHT. There is a significant relationship regarding PHT (p: 0.016).

**Conclusion:**

This study explores the relationship between M2BPGi and systemic involvements in SSc. It demonstrates a significant relationship between M2BPGi and PAP and PHT, suggesting that M2BPGi might serve as a noninvasive biomarker for predicting both PAP and PHT.

## 1. Introduction

Mac-2 binding protein (M2BP, also known as galectin-3 (Gal-3) binding protein) is a highly glycosylated glycoprotein. M2BP serves as a ligand for Gal-3 (formerly known as Mac-2). M2BP has been detected in many body fluids at picogram/mL concentrations and has been shown in studies to perform various functions, including cell adhesion [1]. M2BP interacts with extracellular proteins, including fibronectin, collagen, integrin, inducing fibrosis and activating the immune system. It is thought to contribute to the organism’s cellular response during infections and cancer [2]. High levels of M2BP have been observed especially in melanoma, breast, lung, and gastrointestinal tract cancers, as well as in viral infections. It has been suggested that M2BP may play an important role in the diagnosis and prognosis of these conditions [3]. In addition, Gal-3 has been shown to activate monocytes and macrophages, while epithelial cell-associated Gal-3 plays an active role in the secretion of proinflammatory cytokines [4].

Glycoscience is a newer field within molecular science, and advancements in glycotechnologies are gaining significant importance. Recent studies in glycotechnologies have shown that specific glycan structures and N-glycosylation of M2BP change as liver fibrosis progresses [5]. In recent years, it has been suggested that Wisteria floribunda agglutinin (WFA)+-M2BP, which detects changes in glycans on the surface of M2BP, may serve as a reliable biomarker for liver fibrosis [6]. M2BP glycosylation isomer (M2BPGi), also known as WFA-positive M2BP, regulates the interaction between hepatocytes and Kupffer cells in the context of fibrosis through Mac-2 and is secreted by hepatic stellate cells [7]. M2BPGi is thought to contribute to various inflammatory processes and has been shown in studies to increase during the progression of chronic inflammatory diseases [8]. M2BPGi is also synthesized by cells in the lung. Recent studies suggest that elevated levels of this molecule could be used as a biomarker for idiopathic pulmonary fibrosis (IPF) [9].

Systemic sclerosis (SSc) is a rare, chronic autoimmune connective tissue disease characterized primarily by thickening and hardening of the skin. The pathogenesis involves fibroblast activation and extracellular matrix synthesis, along with vascular remodeling and hyperactivity. This condition leads to fibrosis in the skin and other organs, accompanied by immune system dysfunction [10]. Raynaud’s phenomenon is the most frequently observed clinical manifestation of SSc. Nailfold capillary changes, as well as involvement of the lungs, gastrointestinal system (GIS), and kidneys, are also commonly described. SSc patients are clinically classified into limited cutaneous and diffuse cutaneous forms. This classification is important because diffuse SSc generally has a worse prognosis compared to limited cutaneous SSc [11].

The course of SSc is associated with significant morbidity and mortality. Therefore, early diagnosis and treatment are crucial for patients. Cutaneous symptoms and clinical manifestations play a key role in diagnosis because cutaneous symptoms are the earliest, most common, and most characteristic signs of the disease [12]. Interstitial lung disease (ILD) is leading cause of death associated with SSc. Treatment options are currently limited to addressing the affected organ. Recently, there has been increasing focus on understanding the role of the innate and adaptive immune systems in the disease, with some studies exploring cytokines as targets. However, this blockade has not yet proven successful in treating ILD [13]. Antifibrotic agents effective for lung involvement have not been successful for cutaneous symptoms, increasing the need for biomarkers that are associated with SSc and can be studied for diagnosis and treatment [14]. In relation to Gal-3 and pulmonary hypertension (PHT), it has been proposed that Gal-3 promotes the generation of reactive oxygen species, inflammation, and vascular fibrosis. Additionally, increased levels of Gal-3 were associated with elevated pulmonary arterial pressure (PAP) in various etiologies. Galectin-3 is believed to contribute to PHT through multiple mechanisms [15].

Based on the immune-activating properties of M2BPGi and Gal-3, it was thought that these molecules might be valuable for investigation in SSc. This study aimed to explore the role of M2BPGi serum levels as a biomarker that could contribute to understanding organ involvement and the overall disease process in SSc.

## 2. Materials and methods

The cross-sectional study protocol was approved by the local ethics committee at the board meeting on 28.05.2024, with decision number 2024.100.05.08. The study examined 108 patients with SSc, including 100 women and 8 men, all over the age of 18. These patients were seen at the rheumatology outpatient clinic between 01.06.2024 and 01.09.2024 and were diagnosed with SSc according to the European League Against Rheumatism and American College of Rheumatology classification criteria [16]. In addition, 72 people, 66 women and 6 men, who did not have any systemic disease, malignancy or autoimmune disease, were included in the control group. Patients with a history of other autoimmune diseases, chronic infections, malignancies, or incomplete clinical or laboratory records were excluded. Additionally, pregnant or lactating women, patients with chronic kidney disease, patients using antifibrotic treatments, and individuals under 18 years of age were not included. Demographic and clinical characteristics of the patients, laboratory and radiological findings, pulmonary function tests (PFT) and echocardiography results, presence of PHT based on echocardiographic evaluation, concomitant diseases, and the treatments they received for these were recorded from the hospital’s electronic system, face-to-face patient interviews and outpatient clinic notes. Echocardiography was used to estimate the probability of pulmonary hypertension (PH) following the guidelines of the British Society of Echocardiography (BSE). This approach combines peak tricuspid regurgitant velocity (TRV) measurements with specific echocardiographic markers across three categories: ventricular dimensions, pulmonary artery measurements, and right atrium and inferior vena cava (IVC) characteristics. A TRV measurement above 3.4 m/s generally indicates a high likelihood of PH, while lower TRV values require additional markers to assess PH probability. Key markers include the right ventricular to left ventricular diameter ratio, pulmonary artery diameter, right atrial area, IVC size, and inspiratory collapse. When at least two markers from these categories are present, the likelihood of PH by echocardiography increases [17]. Pulmonary artery pressures of 10 patients who underwent right heart catheterization (RHC) were also recorded. The presence and degree of skin fibrosis was evaluated using the modified Rodnan skin score (mRSS), and ILD was evaluated with high-resolution lung tomography (HRCT) findings. SSc activity was calculated with The European Scleroderma Study Group (EScSG) activity scoring system [18] and United Kingdom (UK) Functional Activity Score, and patients with a score ≥3 were defined as active patients.

Venous blood samples of 5 mL were collected from individuals in both the patient and control groups into gel biochemistry tubes after 8–12 h of fasting. The samples were centrifuged at 3000 RPM for 10 min, and the serum portion was separated and stored at −80 °C until analysis. Prior to analysis, the samples were brought to room temperature and vortexed. Human M2BPGi levels in the samples were measured using the SunRed brand ELISA kit (Catalog No. 201-12-6894), which detects M2BPGi levels by the double antibody sandwich technique. The lower limit of detection for this ELISA kit is 8.628 pg/mL, with an intra-assay coefficient of variation (CV) of <10% and an inter-assay CV of <12%.

After measuring M2BPGi levels in the patient and control groups, the statistical relationships between these levels and factors such as disease activity, commonly associated antibodies in the course of SSc, systemic involvement patterns, and the EScSG score were analyzed.

Statistical analysis for the study was performed using Statistical Package for the Social Sciences-SPSS 27 software with a 95% confidence interval. T-tests, ANOVA, chi-square tests, and correlation tests were used in the analyses. Specifically, M2BPGi levels were analyzed using t-tests for variables with two groups and ANOVA for variables with three or more groups. The chi-square test was employed to examine the relationship between group and gender. Pearson correlation tests were used to assess the relationship between M2BPGi levels and other measurements. Logistic regression analysis was conducted to adjust for age, sex, and comorbidities.

## 3. Results

In the study, 108 patients with SSc and 72 control patients were examined. In the SSc group, 100 (92.6%) were women and 8 (7.4%) were men. In the control group, 66 (91.7%) were women and 6 (8.3%) were men. There was no significant difference in gender distribution between the two groups (p = 1.000). The mean age of the SSc group was 56.04 ± 10.47 years, while the mean age of the control group was 53.89 ± 7.96 years. No statistically significant difference in age distribution was found between the groups (p = 0.299). Among the patients, 34 (31.5%) were smokers, 6 were ex-smokers, and 68 were nonsmokers. Regarding SSc involvement, 46 patients had diffuse SSc and 62 had limited SSc.

When evaluating the patient group in terms of family history, 8 patients had a family history of SSc, and 16 had a family history of other rheumatic diseases, totaling 22.2%. Regarding comorbid conditions, the following diagnoses were noted: hypertension in 36 patients, diabetes mellitus in 22 patients, hyperlipidemia in 8 patients, coronary artery disease in 8 patients. Examining the treatments used by the patients, the following was observed: bosentan and azathioprine were each used by 16 patients (14.8%), colchicine was used by 52 (48.1%), pentoxifylline by 44 (40.7%), and phosphodiesterase inhibitors by 16 patients (14.8%). Cyclophosphamide was used by 8 (7.4%), rituximab by 2 (1.9%), and corticosteroids by 52 patients (48.1%). Iloprost was used by 20 patients (18.5%), calcium channel blockers by 84 (77.8%), hydroxychloroquine by 98 (90.7%), and acetylsalicylic acid by 94 patients (87%). Mycophenolate was used by 28 patients (25.9%), methotrexate by 46 (42.6%), and proton pump inhibitors by 66 patients (61.1%). Additionally, 26 of them (24.1%) used angiotensin receptor blockers or angiotensin-converting enzyme inhibitors.

Disease activity, laboratory findings, antibody positivity profiles, respiratory function test results, HRCT, and echocardiography findings were examined. Disease activity and functional evaluations, including the modified Rodnan score, EScSG activity index, and UK functional scoring, showed no statistically significant relationship with M2BPGi levels (p > 0.05 for all) (As shown in Table 1 and Table 2). When examining the patient group for organ and system involvement, the following was observed: GIS involvement in 64 patients (59.3%), HRCT involvement (patients with ILD findings on HRCT) in 42 (38.9%), cardiac involvement (reduced ejection fraction or abnormal right ventricular function, valve disease, or other echocardiographic findings not attributable to other causes) in 24 (22.2%), and renal involvement (reduced glomerular filtration rate) in 22 patients (20.4%). No cases of SSc renal crisis were identified. Increased PAP, as evaluated by echocardiography, was found in 34 patients (31.5%), and PHT (patients with high PAP were considered to have PHT) [17] was detected in 34 patients.

There was no significant difference between the M2BPGi levels in the patient group (median = 4749.69 pg/mL, mean = 5351.75 ± 2483.97) and the control group (median = 4638.07, mean = 4611.86 ± 1333.15) (p = 0.071). Overall, the M2BPGi level for both groups combined was measured with a median of 4697.14 and a mean of 5055.8 ± 2122.76 pg/mL (As shown in Table 3). Although M2BPGi levels did not reach statistical significance in the SSc group, they were found to be higher than in the control group. When examining the relationship between M2BPGi and laboratory parameters, no significant relationships were detected with disease age (p = 0.279), disease type (p = 0.359), mRSS (p = 0.869), EScSG activity index (p = 0.639), or UK functional scoring (p = 0.643). Additionally, no relationship was found between the patients’ ejection fractions (EFs), forced vital capacities (FVCs), diffusing capacities of the lungs for carbon monoxide (DLCOs), and M2BPGi levels (p = 0.298, p = 0.451, p = 0.182, respectively) (as shown in Table 1).

M2BPGi levels did not show significant variation between the limited SSc group (median = 4795.7, mean = 5762.49 ± 2938.04) and the diffuse SSc group (median = 4493.89, mean = 4893.72 ± 1743.9) (p = 0.359). Similarly, there was no significant difference between patients with comorbid diseases (median = 4749.69, mean = 5002.14 ± 1924.5) and those without (median = 4631.71, mean = 5788.76 ± 3031.4) (p = 0.251). The levels also did not differ significantly between the passive smoking group (median = 4467.71, mean = 5304.16 ± 2611.51) and the active smoking group (median = 5029.59, mean = 5652.92 ± 2270.8) (p = 0.662). Additionally, no significant difference was observed between individuals with a family history of SSc (median = 4785) and those without (median = 4448.1, mean = 5256.45 ± 2560.02) (p = 0.882).

The analysis of drug use in relation to M2BPGi levels revealed a significant difference between patients who did not use methotrexate (MTX) (median = 5065.89, mean = 5898.68 ± 3054.92) and those who used MTX (median = 4467.71, mean = 4614.59 ± 1071.69) (p = 0.036). No significant relationship was found between M2BPGi levels and the use of other medications (p > 0.05).

No significant difference in M2BPGi levels was observed between patients with or without lung involvement detected by HRCT (median = 4723.4 vs. 4795.7; mean = 5589.49 ± 2814.24 vs. 4978.17 ± 1854.45) (p = 0.383). Similarly, levels did not differ significantly between those with or without GIS involvement (median = 4884.63 vs. 4428.5; mean = 5992.81 ± 3024.42 vs. 4911.03 ± 1964.76) (p = 0.117). There was no significant difference in levels between patients with or without cardiac involvement (median = 4608.65 vs. 5089.08; mean = 5291.39 ± 2434.91 vs. 5563.03 ± 2750.95) (p = 0.742). Additionally, no significant difference was detected between those with or without kidney involvement (median = 4993 vs. 4128.35; mean = 5576.01 ± 2664.56 vs. 4783.48 ± 1653.4) (p = 0.418).

Considering PAP status, the mean value of systolic PAP detected by echocardiography was 36.7 mmHg and the average M2BPGi level in the normal PAP group was 5898.15 ± 2555.61 pg/mL, while it was 4258.96 ± 1973.08 pg/mL in the increased PAP group. The difference between these two groups was statistically significant (p: 0.021). The mean PAP of 10 patients who underwent RHC was calculated as 37.34 mmHg and the mean M2BPGi value of this subgroup was 4266 pg/mL. This finding indicates that the M2BPGi level decreases significantly as PAP increases. Similarly, when examining PHT status, the average M2BPGi level was 5942.01 ± 2579.14 pg/mL in the group without PHT, decreasing to 4264.44 ± 1917.63 pg/mL in the group with PHT. There is a significant relationship between the two groups regarding PHT (p: 0.016). This result shows that the presence of PHT significantly reduces M2BPGi levels (as shown in Table 4). Logistic regression analysis was conducted to adjust for age, sex, and comorbidities. After these adjustments, the significant associations between M2BPGi levels and both PAP and PHT persisted (p = 0.27 for PAP, p = 0.32 for PHT). The patients were stratified based on antibody profiles, including anti-Scl-70 and anticentromere antibodies, and found that M2BPGi levels were not significantly affected by these factors (p = 0.45 for anti-Scl-70, p = 0.38 for anticentromere).

## 4. Discussion

This study is notable for being the first to investigate M2BPGi levels in the context of SSc. It provides a foundational basis for further research into this glycosylation isomer, which may be associated with the disease and its systemic involvements. To date, no published studies have explored M2BPGi levels specifically within the field of SSc. Although the study did not find a statistically significant correlation between M2BPGi levels in patients with SSc and those in the control group, the data suggests a potential trend. The observed increase in M2BPGi levels within the SSc group indicates that, with a larger patient sample, this difference might have reached statistical significance. The lack of a significant difference in M2BPGi levels between SSc patients and the control group may be attributable to the complex and multifaceted pathophysiology of SSc. This autoimmune condition is characterized by significant heterogeneity in disease progression, organ involvement, and immune response, which could dilute measurable differences in certain biomarkers like M2BPGi when assessed in a cross-sectional setting. Additionally, the role of fibrosis and immune activation, key components of SSc pathogenesis, might not uniformly translate into elevated M2BPGi levels across the patient cohort.

Recent studies have identified M2BPGi, also known as immunoassay-based glycan “sugar chains”, as a promising novel biomarker for predicting liver fibrosis[19]. Studies in the field of oncology have shown that oligomeric glycoprotein 90K, also known as M2BP, is found at higher levels in the serum of some patients with tumors [20]. While many studies have identified a significant relationship between M2BPGi and conditions such as fibrosis, portal hypertension, and cirrhosis, the findings in this study showed that M2BPGi levels in SSc patients were similar to those in the control group, specifically regarding fibrosis-related organ involvement.

Some studies in the literature have examined the relationship between SSc and galectin-3. In one study, 37 SSc patients were compared with a healthy control group. It was found that galectin-3 levels were elevated in SSc patients, with higher levels observed in those with active SSc compared to those with inactive SSc. The study suggested that Gal-3 could serve as a biomarker for SSc activation [21]. Additionally, echocardiography was used to assess PAP, and consistent with our findings, no significant relationship was observed between lung involvement, GIS involvement, and the isomer. It was previously suggested that different molecules and pathways might be confounding the interaction between the newly studied molecule M2BPGi and the previously investigated Gal-3. Other studies have underscored the importance of glycans and glycan-binding proteins in leukocyte capture and rolling mechanisms. For example, endogenous Gal-3, has been shown to enhance the recruitment of neutrophils and lymphocytes in living organisms [22, 23]. In models with Gal-3 deficiency, reduced leukocyte rolling and migration were noted during acute inflammation. Conversely, treatment with recombinant Gal-3 led to a decrease in rolling speed and an increase in neutrophil and monocyte adhesion [23]. Additional in vitro studies support direct influences of Gal-3 on leukocyte movement, suggesting their potential roles in vascular inflammation and immune cell recruitment within SSc [24].

In a study conducted in Japan that examined the relationship between Gal-3 and the SSc process, the levels in patients with diffuse cutaneous SSc were found to be significantly lower than in the control group. It was also noted that Gal-3 levels increase when right ventricular systolic pressure rises [25]. In the current study, no significant relationship was found between the skin score and isomer serum levels. However, the significant relationship between M2BPGi and PAP suggests that further research in this area may be warranted. In a 2024 study that investigated M2BP in patients with PHT, it was reported that M2BP levels increased in PHT and were correlated with the severity of blood pressure [26].

In this study, the relationship between the treatments received by the patients and the serum level of the M2BPGi glycosylation isomer was also examined. The significant relationship observed with MTX suggests that MTX might be the most effective treatment for inflammation in the complex developments during the course of SSc in the studied patient group. In another study, consistent with our findings, a significant decrease in serum levels of Gal-1 and Gal-3 was observed in SSc patients undergoing treatment with MTX [27]. The observed difference in M2BPGi levels between patients using MTX and those not using it might be related to its role in modulating inflammatory and fibrotic processes. While it is true that MTX is primarily effective for managing skin involvement in diffuse SSc, its systemic immunomodulatory effects could contribute to changes in biomarkers, including M2BPGi, even without direct evidence of efficacy on other organ involvements. This indicates that MTX could be one of the effective agents for preventing fibrosis, inflammation, and mixed processes in the SSc process.

This study demonstrated a significant correlation between M2BPGi levels and sPAP in SSc patients. However, the observation that M2BPGi levels were lower in patients with elevated PAP and PHT contrasts with findings reported in the existing literature, and several factors may account for this discrepancy. One possibility is that the demographic and clinical characteristics of the patient population in this study with different baseline M2BPGi levels. Factors such as treatment histories could influence M2BPGi levels and may explain this variation. Measuring the isomer level while patients were undergoing treatment, and the inclusion of drugs added to SSc treatment in those with PHT or increased arterial pressure, may have altered the isomer level. Additionally, methodological differences between studies could play a role. Another factor to consider is the duration and severity of PHT in the study population. Mechanisms beyond inflammation may contribute to the increase in PAP and PHT in SSc[28]. Proposed mechanisms include endothelial damage, vascular remodeling, vasoconstriction, genetic polymorphisms, abnormal vascular responses due to interstitial lung damage, and vascular stiffness [29]. Despite these variations, the literature and this study’s findings suggest that M2BPGi glycosylation isomer holds potential as a useful biomarker for indicating PAP and PHT in SSc patients, warranting further exploration.

No significant relationship was found between disease activity indices and the isomer, suggesting that while M2BPGi may retain importance as a biomarker reflecting certain organ involvements in SSc, its association with disease activity and functional status might be limited. The lack of a significant association between M2BPGi levels and pulmonary function parameters, such as FVC and DLCO, suggests that M2BPGi may not directly reflect lung function impairment. While M2BPGi is considered a marker of fibrosis and immune activation, its role may be more specific to certain systemic involvements rather than generalized pulmonary dysfunction. This finding indicates that M2BPGi’s potential as a biomarker could be more relevant for assessing vascular or other organ-specific involvements in SSc, rather than direct measures of lung function.

When PAPs were evaluated in the subgroup of patients who underwent RHC, it was observed that patients with elevated PAP had significantly lower M2BPGi levels compared to those with normal PAP. This finding aligns with noninvasive measurement data, suggesting that M2BPGi could serve as a potential biomarker for PHT presence. The additional RHC data strengthens the reliability of our findings, highlighting the possible role of M2BPGi in the diagnosis of SSc-related PHT. The lack of change in isomer levels across antibody stratifications suggests that M2BPGi may serve as a biomarker independent of specific antibody profiles in SSc.

In conclusion, this study explores the relationship between M2BPGi, a molecule increasingly studied worldwide, and fibrosis and systemic involvements in SSc. It demonstrates a significant relationship between M2BPGi and PAP and PHT, suggesting that M2BPGi might serve as a noninvasive biomarker for predicting both PAP and PHT.

This study has certain limitations that should be acknowledged. Firstly, the use of echocardiography as a diagnostic tool for PHT may not provide the accuracy of RHC, which remains the gold standard. While echocardiography offers a noninvasive approach, it may lack the precision required for definitive PHT diagnosis. Secondly, the relatively small sample size may limit the generalizability of our findings. Additionally, the cross-sectional design of the study restricts our ability to infer causality between M2BPGi levels and disease progression. Future studies with larger, longitudinal cohorts, including echocardiographic assessments of disease-free control groups, are necessary to validate these findings and to explore the potential of M2BPGi as a biomarker in SSc. These studies should ideally exclude patients currently using medications that might influence M2BPGi levels, carefully adjust for the duration and severity of PHT, and account for methodological and ethnic differences to ensure the robustness and generalizability of the findings.

## Figures and Tables

**Table 1:** Relationship between M2BPGi levels and clinical disease activity, demographic, clinical parameters and functional evaluation of patients (HRCT findings, PFT results, and echocardiography findings) diagnosed with SSc.

Clinical disease activity, demographic and clinical parameters and functional evaluation for patients		Relationship with M2BPGi level (p values)*	r values
Modified Rodnan score mean (SD)	9.13(9.23)	*0.869*	*0.14*
EScSG activity index mean (SD)	3.89(1.99)	*0.639*	−*0.47*
UK functional scoring mean (SD)	10.87(9.84)	*0.643*	−*0.22*
Disease duration (SD)	10.86(7.91)	*0.279*	−*0.21*
Disease type (diffuse SSc, n (%) and limited SSc, n(%))	46(42.6) 62(57.4)	*0.359*	−*0.28*
GIS involvement, n (%)	64 (59.3)		
Cardiac involvement, n(%)	24 (22.2)		
Renal involvement, n (%)	22 (20.4)		
**PFT, HRCT, PAB, echocardiographic findings**			
FVC mean(SD) (normal:> 80)	92.13(2.96)	*0.182*	
DLCO mean(SD) (normal: 60–120)	70.79(25.09)	*0.451*	*0.24*
EF mean(SD)	58.61(8.51)	*0.298*	*0.18*
PAP increase n(%)	34 (31.5)	***0.021***	−*0.39*
HRCT findings n(%)	42 (38.9)	*0.383*	*0.08*
sPAP by tricuspid regurgitation (TR) peak velocity (PHT is estimated if TR > 2.8 m/s)	34 (31.5)	***0.016***	−*0.40*

* Statistical analysis made by Pearson correlation analysis

**Table 2:** Relationship between M2BPGi levels and laboratory values of patients diagnosed with SSc.

Laboratory values of patients		Relationship with M2BPGi level (p values)*	r values
Hb (g/dL)	12.25(1.32)	*0.727*	*0.18*
WBC (1000/μL)	6.28(1.81)	*0.421*	*0.39*
PLT (1000/μL)	241.37(86.19)	*0.855*	−*0.41*
Pro BNP (pg/dL)	163.44(82.37)	*0.462*	*0.08*
**Antibody profile**	**n(%)**		
Antinuclear antibody		*0.292*	−*0.47*
ANA (1+)	4 (7.4)		
ANA (2+)	6 (11.1)		
ANA (3+)	11 (20.4)		
ANA (4+)	33 (61.1)		
Antitopoisomerase	25 (46.3)		
Anti Ro	10 (18.5)		
Anti La	3 (5.6)		
Anticentromer	31 (57.4)		
RNP	2 (3.7)		

* Statistical analysis made by Pearson correlation analysis.

**Table 3:** M2BPGi levels (pg/mL) in the patient group, control group, and overall, along with the interpretation of the statistical relationships between the groups.

M2BPGi level	Groups						*p*
	SSc patients		Control group		Total		
	(The lowest-highest values) Median	Mean(SD)	(The lowest-highest values) Median	Mean (SD)	(The lowest-highest values) Median	Mean (SD)	
**pg/mL**	(1436.92–15,075) 4749.69	5351.75(2483.97)	(598.12–8904.91) 4638.07	4611.86 (1333.15)	(598.12–15,075) 4697.14	5055.8 (2122.76)	*0.071*

**Table 4:** The relationship between organ and system involvement and M2BPGi levels.

System involvements		(The lowest–highest values) Median	Mean(SD)	*p*
Lung involvement by HRCT^a^	Absent	(2813.71–15075) 4723.4	5589.49(2814.24)	*0,383*
	Present	(1436.92–9469.35) 4795.7	4978.17(1854.45)	
Pulmonary arterial pressure (PAP)^a^	Normal	(3545.13–15075) 5065.89	5872.09(2524.84)	***0,021***
	Increased	(1436.92–9469.35) 3596.77	4219.25(2026.37)	
Pulmonary hypertension (PHT)^a^	Absent	(2813.71–15075) 5065.89	5773.43(2606.44)	***0,016***
	Present	(1436.92–9469.35) 4095.82	4433.99(1961.33)	
Gastrointestinal involvement^a^	Absent	(2832.86–15075) 4884.63	5992.81(3024.42)	*0,117*
	Present	(1436.92–10817.14) 4428.5	4911.03(1964.76)	
Cardiac involvement^a^	Absent	(1769.07–15075) 4608.65	5291.39(2434.91)	*0,742*
	Present	(1436.92–10817.14) 5089.08	5563.03(2750.95)	
Renal involvement^b^	Absent	(1436.92–15075) 4993.29	5576.01(2664.56)	*0,418*
	Present	(3120.7–7392.88) 4030.83	4359.44(1327.84)	

a = t test;

b = ANOVA test
